# Epoxyeicosatrienoic acid analog attenuates angiotensin II hypertension and kidney injury

**DOI:** 10.3389/fphar.2014.00216

**Published:** 2014-09-23

**Authors:** Md. Abdul Hye Khan, John R. Falck, Vijaya L. Manthati, William B. Campbell, John D. Imig

**Affiliations:** ^1^Department of Pharmacology and Toxicology, Medical College of WisconsinMilwaukee, WI, USA; ^2^Department of Biochemistry, University of Texas Southwestern Medical CenterDallas, TX, USA; ^3^Cardiovascular Research Center, Medical College of WisconsinMilwaukee, WI, USA

**Keywords:** epoxyeicosatrienoic acid analog, mesenteric artery, endothelial function, renal inflammation, angiotensin II

## Abstract

Epoxyeicosatrienoic acids (EETs) contribute to blood pressure regulation leading to the concept that EETs can be therapeutically targeted for hypertension and the associated end organ damage. In the present study, we investigated anti-hypertensive and kidney protective actions of an EET analog, EET-B in angiotensin II (ANG II)-induced hypertension. EET-B was administered in drinking water for 14 days (10 mg/kg/d) and resulted in a decreased blood pressure elevation in ANG II hypertension. At the end of the two-week period, blood pressure was 30 mmHg lower in EET analog-treated ANG II hypertensive rats. The vasodilation of mesenteric resistance arteries to acetylcholine was impaired in ANG II hypertension; however, it was improved with EET-B treatment. Further, EET-B protected the kidney in ANG II hypertension as evidenced by a marked 90% decrease in albuminuria and 54% decrease in nephrinuria. Kidney histology demonstrated a decrease in renal tubular cast formation in EET analog-treated hypertensive rats. In ANG II hypertension, EET-B treatment markedly lowered renal inflammation. Urinary monocyte chemoattractant protein-1 excretion was decreased by 55% and kidney macrophage infiltration was reduced by 52% with EET-B treatment. Overall, our results demonstrate that EET-B has anti-hypertensive properties, improves vascular function, and decreases renal inflammation and injury in ANG II hypertension.

## INTRODUCTION

Cytochrome P450 epoxygenase metabolites of arachidonic acid, epoxyeicosatrienoic acids (EETs), have numerous cardiovascular and renal actions that make them therapeutically promising in cardiovascular and renal diseases (Imig, 2008, 2010, 2012; Imig and Hammock, 2009; Sudhahar et al., 2010). Accordingly, inhibition of soluble epoxide hydrolase (sEH), which hydrolyzes EETs to their less biologically active dihydroxyeicosatrienoic acid metabolites, increases EETs bioavailability and exhibited therapeutically important cardiovascular effects (Imig et al., 2002, 2005; Imig and Hammock, 2009; Imig, 2010, 2012).

In line with the observations that increased EET bioavailability by sEH inhibition provides beneficial cardiovascular and renal effects, several sEH inhibitors have been developed and tested for anti-hypertensive effects in multiple models of hypertension including angiotensin (ANG II)-dependent hypertension (Imig et al., 2002, 2005; Zhao et al., 2004; Loch et al., 2007; Manhiani et al., 2009). Apart from sEH inhibitors, one relevant approach to therapeutically targeting EETs for cardiovascular diseases would be the development of agonist analogs for EETs. This is an important approach as biological effects of EETs are limited by solubility and storage issues (Falck et al., 2003). EET analogs are designed to resist metabolism and improve solubility (Yang et al., 2007; Falck et al., 2009). Moreover, several earlier studies demonstrated that these EET analogs vasodilate coronary, renal, and mesenteric arteries (Imig et al., 1999; Gauthier et al., 2004; Dimitropoulou et al., 2007). Considering the promising vascular actions of early generation EET analogs, it was important to develop orally active EET analogs, which can eventually be developed as novel cardiovascular therapeutic agents. Indeed, a number of experimental studies support the use of EET analogs in cardiovascular disease. An EET analog, NUDSA, dilated renal afferent arterioles, reduced blood pressure in hypertensive rats and mice, improved insulin sensitivity in metabolic syndrome mice, and decreased experimental cardiac reperfusion injury (Seubert et al., 2007; Sodhi et al., 2009; Imig et al., 2010).

More recently, newer generations of EET analogs have been developed and are beginning to demonstrate therapeutic potential when administered *in vivo* either acutely or chronically. We have developed a series of orally active EET analogs and demonstrated their anti-hypertensive and organ protective effects in a number of pathologies (Hye Khan et al., 2013, 2014). In the present study, we investigated the anti-hypertensive effect of one such EET analog, EET-B, synthesized by replacing the COOH group at carbon 1 of the EET pharmacophore with a heterocyclic surrogate. We demonstrate anti-hypertensive and kidney protective effects of this EET analog in ANG II induced hypertension.

## MATERIALS AND METHODS

### CHEMICALS

**FIGURE 1:**
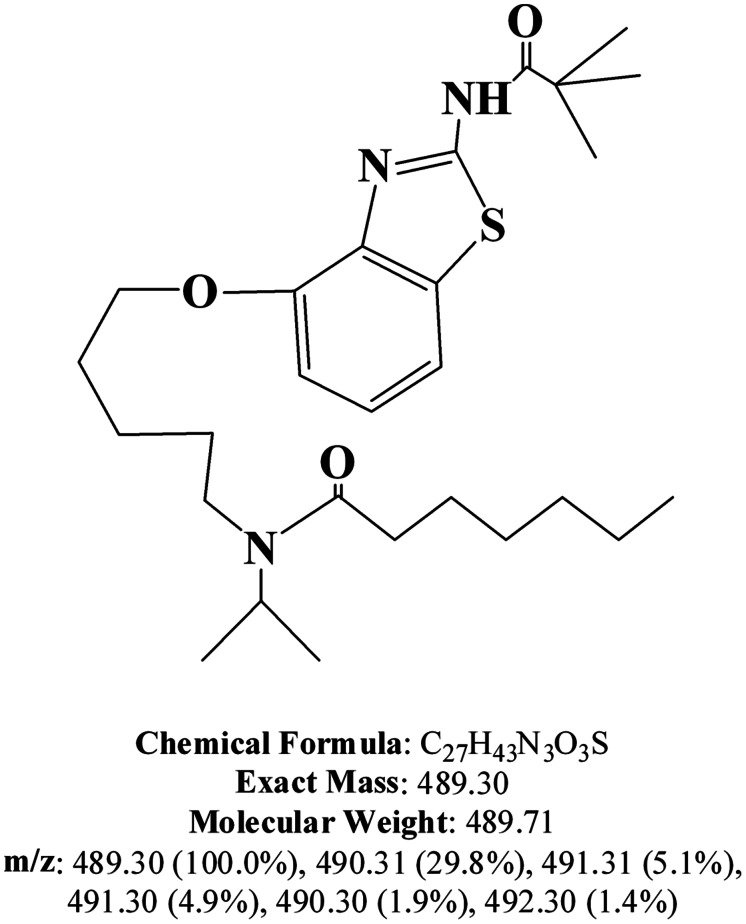
**Structure of EET-B [(*N*-(5-((2-acetamidobenzo[d]thiazol-4-yl)oxy) pentyl)-*N*-isopropylheptanamide)]**.

All chemicals were purchased from Sigma-Aldrich (Ann Arbor, MI, USA). The EET analog, EET-B [(*N*-(5-((2-acetamidobenzo[d]thiazol-4-yl)oxy) pentyl)-*N*-isopropylheptanamide)] was designed, synthesized, and analyzed for purity in the laboratory of John R. Falck. The structure of EET-B is described in **Figure 1**.

### ANIMALS

Male Sprague-Dawley (SD) rats weighing between 225 and 275 g (*n* = 6–8/group) and purchased from Charles River Laboratories were used in this study. Animal protocols were in accordance with National Institutes of Health guidelines and approved by the Institutional Animal Care and Use Committees. Animals were fed normal rat chow throughout the experiment and were housed under conditions of constant temperature and humidity with a 12:12 h light–dark cycle. Rats were allowed to acclimatize to these conditions for 7 days before starting any experimental protocol.

### TELEMETRY BLOOD PRESSURE MEASUREMENT

In order to measure blood pressure, telemetry transmitters (Data Sciences Inc., Saint Paul, MN, USA) were implanted 2 weeks prior to the experiment in rats under isoflurane anesthesia. A subcutaneous pocket between the caudal edge of the flank and the most cranial extension of the knee’s range of motion was made. The transmitter body was placed in the subcutaneous pocket with the tissue snug. The transmitter catheter inserted into femoral artery and advanced to the abdominal aorta. The incision was closed with sutures and sealed with sterile tissue adhesive to help prevent infection. The suture was removed 7 days later after the incision had completely healed. Rats were allowed to recover from surgery and were returned to individual housing. A baseline arterial pressure was recorded for 3–5 days prior to the experimental period. Blood pressure and heart rate were continuously recorded throughout the experimental period.

### ANTI-HYPERTENSIVE EFFECTS OF EET-B IN ANG II-HYPERTENSIVE RATS

This experiment was carried out in a set of rats implanted with an ANG II filled ALZET®;mini-osmotic pumps (s.c.) to deliver ANG II at a dose of 60 ng/min for 14 days and blood pressure monitored by telemetry transmitter. On the first day of ANG II pump implantation, EET-B treatment in drinking water *ad libitum* was started and continued for 14 days while blood pressure and heart rate were monitored. EET-B, administered in drinking water (10 mg/kg/day), was prepared with 1% PEG-400 and 0.05% ethanol. The vehicle-treated groups received drinking water containing 1% PEG-400 and 0.05% ethanol *ad libitum* and blood pressure and heart rate were monitored.

### VASCULAR REACTIVITY STUDIES

Second order mesenteric resistance arteries were collected at the end of the experimental protocol to determine vascular function. Arterial segments were mounted between two glass cannulas in a pressure myograph system (Danish Myo Technology model 111P, DMT, Aarhus, Denmark). Mesenteric arteries were oxygenated in 95% O_2_/5% CO_2_ Krebs physiological salt solution at pH 7.4 and 37°C. Under no-flow conditions, the pressure within the vessel was increased in 10-mmHg increments from 20 to 65 mmHg. The blood vessel was then equilibrated at 65 mmHg for 30 min and control lumen diameter was calculated as the mean diameter during the last minute of the 30-min equilibration period. Mesenteric resistance arteries were constricted with the thromboxane mimetic U-46619 and diameter of the constricted artery was calculated as the mean during the last minute of the 15 min period. Following U46619 constriction, vessel diameter responses to graded concentrations of acetylcholine (0.001–10 μM) were assessed. The nitric oxide donor, sodium nitroprusside (100 μM) was added to the bath at the end of the experimental period to ensure vascular integrity. Relaxation responses were plotted as a percent relaxation from maximum contraction.

### BIOCHEMICAL MEASUREMENTS

Urinary biochemical analysis was done using commercially available ELISA assay kits; albumin and nephrin from Exocell (Philadelphia, PA, USA), and monocyte chemoattractant protein-1 (MCP-1) from BD Biosciences (San Jose, CA, USA). Urinary sodium was measured using ISE (Ion Selective Electrode) based method (EasyLyte Analyzer, Medica Corporation, Bedford, MA, USA).

### KIDNEY HISTOLOGY

For histological analysis, kidneys were excised, longitudinally sectioned, immersion-fixed in 10% neutral buffered formalin and paraffin-embedded. The kidney sections were embedded and cut into 4 μm slices for use in histology protocols. Formalin-fixed paraffin-embedded kidney slices were de-paraffinized, re-hydrated and stained with Periodic Acid-Schiff (PAS) reagents. Two observers in a blinded design conducted histological analysis of tubular casts in the PAS stained slides at a magnification of 200x using Nikon NIS Elements Software. The percentage area positive for cast was calculated from the mean of 10 fields (200x) for each animal.

### IMMUNOHISTOPATHOLOGICAL ANALYSIS

Formalin-fixed and paraffin-embedded kidney slices were de-paraffinized, re-hydrated, and subjected to immunohistochemistry protocol. Kidney sections were immunostained with anti-CD68 (1:50; Serotec, Raleigh, NC, USA) to determine macrophage/monocyte infiltration. Biotinylated donkey anti-mouse secondary antibody (1:200) was used for development with avidin-biotinylated HRP complex (Vectastain ABC Elite kit, Vector Laboratories, Burlingame, CA, USA) followed by counterstaining with hematoxylin and mounted. Stained sections were visualized by light microscopy at 400x magnification and digital images of the stained kidney sections were taken for analysis using Nikon NIS Elements Software (Nikon Instruments Inc., Melville, NY, USA). Macrophage/monocyte infiltration was determined by point counting of CD68-positive cells by two experienced reviewers in a blinded design. Twenty images were captured for each kidney section and the number of positive cells per image was divided by the metric area of the image to obtain the number of positive cells per mm^2^.

### STATISTICS

Data expressed as mean ± S.E.M. Statistical significance between two measurements was determined by the two-tailed unpaired Student’s *t-*test (and among groups it was determined by repeated measure one-way analysis of variance followed by Tukey’s *post hoc* test) using GraphPad Prism®;Version 4.0 software (GraphPad Software Inc, La Jolla, CA, USA). Probability values of *P* < 0.05 were considered significant where the critical value of *P* was two-sided.

## RESULTS

### EET-B REDUCES BLOOD PRESSURE IN ANG II HYPERTENSIVE RATS

**FIGURE 2:**
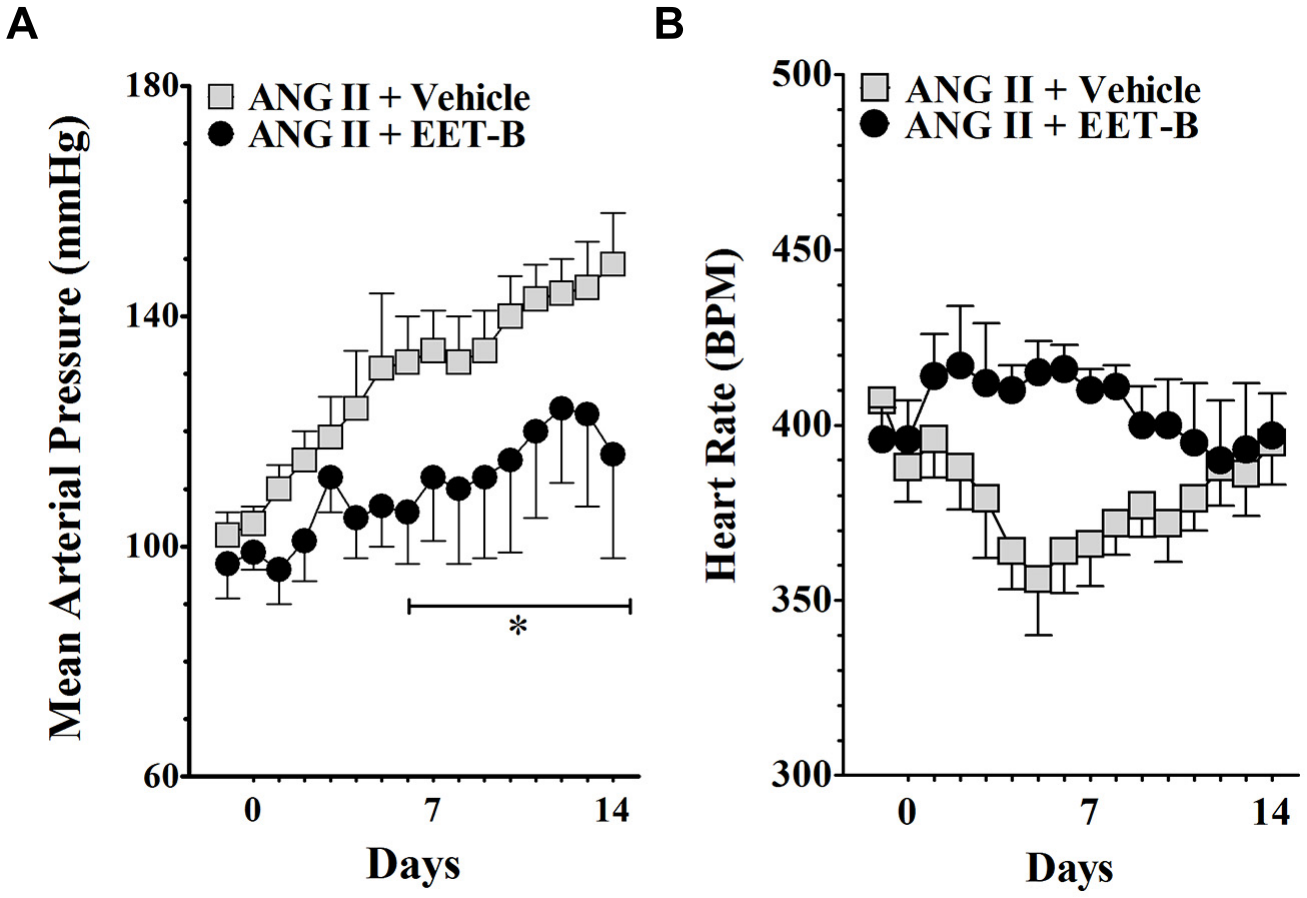
**Effects of orally administered EET-B on mean arterial pressure and heart rate in angiotensin II (ANG II) hypertensive rats.** EET-B was administered at a dose of 10 mg/kg/day in the drinking water for 14 days. Blood pressure **(A)** and heart rate **(B)** data are presented as the average of 24 h readings. *P < 0.05 vs. ANG II + Vehicle. All data presented as mean ± SEM, *n* = 8/group.

As demonstrated in **Figure 2**, oral administration of EET-B markedly blunted the development of ANG II hypertension without affecting heart rate. At the end of the 14-day ANG II infusion period, mean arterial pressure in vehicle-treated ANG II hypertensive rats averaged 152 ± 4 mmHg. EET-B treated ANG II hypertensive rats had lower blood pressure at the end of 14-day period and mean arterial pressure averaged 114 ± 14 mmHg (*P* < 0.05; **Figure 2A**). Heart rate was not different between vehicle and EET-B treatment in ANG II hypertensive rats. The heart rate averaged 419 ± 8 and 400 ± 19 beat/min in vehicle and EET-B-treated ANG II hypertensive rats, respectively (**Figure 2B**). Urinary sodium excretion on day 14 was similar between EET-B (2.2 ± 0.3 mmol/d) and vehicle (2.7 ± 0.4 mmol/d) treated ANG II hypertensive rats. Moreover, at the end of the 14-day treatment period there was no difference in the daily urine volume of ANG II hypertensive rats treated with vehicle or EET-B (32 ± 4 vs. 29 ± 5 ml/day).

### EET-B PRESERVES VASCULAR FUNCTION IN ANG II HYPERTENSION

**FIGURE 3:**
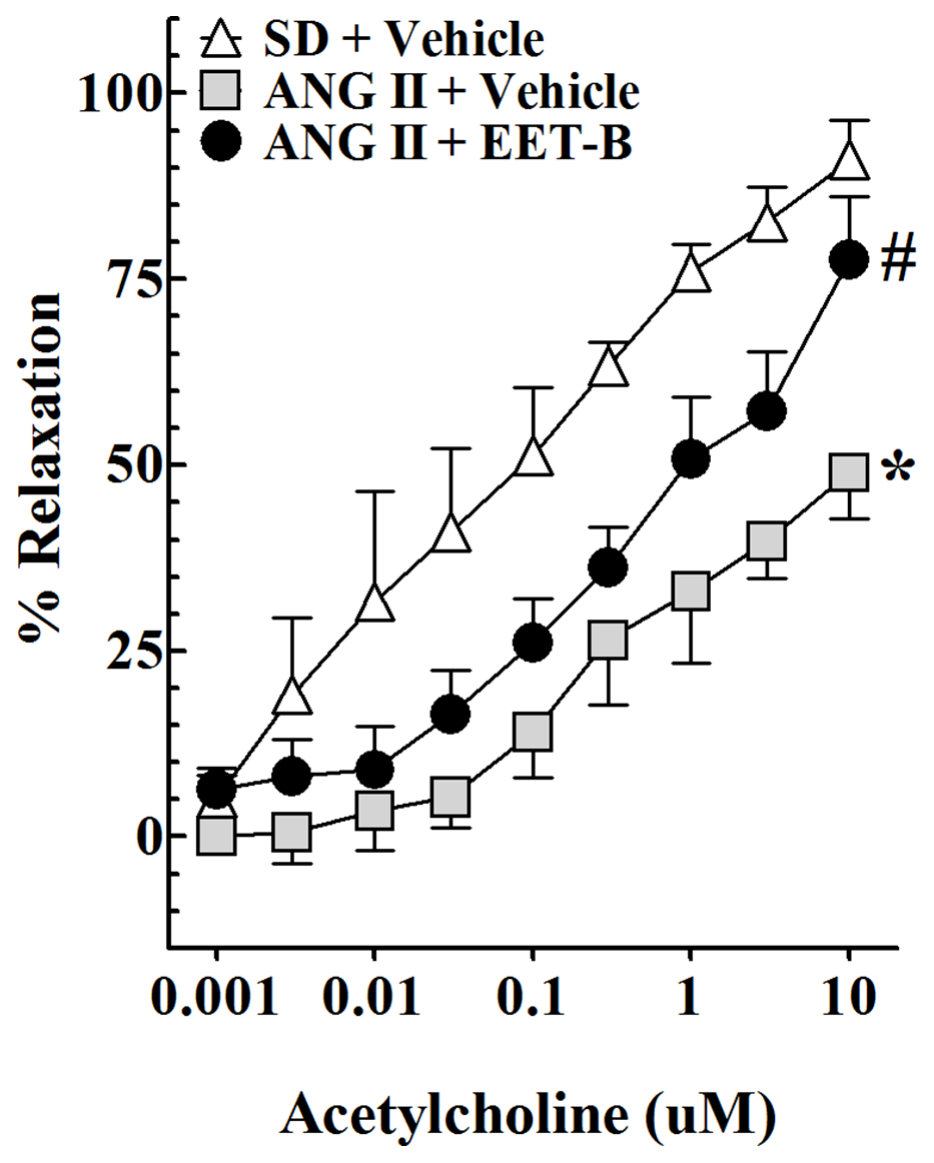
**Effect of orally administered EET-B on mesenteric resistance artery function in angiotensin II (ANG II) hypertension.** EET-B was administered at a dose of 10 mg/kg/day in the drinking water for 14 days. Mesenteric resistance artery basal diameter was not different between experimental groups averaging 288 ± 37 μm (*n* = 24) under control conditions and 131 ± 14 μm after administration of the thromboxane mimetic U46619. Mesenteric resistance artery responses to acetylcholine (0.001–10 μM) were determined. *P < 0.05 vs. Sprague-Dailey (SD) + Vehicle and #*P* < 0.05 vs. ANG II + Vehicle. All data presented as mean ± SEM, *n* = 8/group.

In the present study, we determined the vasorelaxation response of mesenteric resistance arteries to acetylcholine in vehicle and EET-B-treated ANG II hypertensive rats at the end of 14-day experimental protocol. Among the experimental groups, there were no differences in the baseline arterial diameters (288 ± 37 μm) or diameters after treatment with the thromboxane mimetic U46619 (131 ± 14 μm). Relaxation of mesenteric resistance arteries to acetylcholine was significantly blunted in ANG II hypertension compared to control SD rats (*P* < 0.05). Mesenteric artery relaxation to 10 μM acetylcholine averaged 91 ± 2% in control SD and 49 ± 3% in ANG II hypertensive rats (**Figure 3**). Interestingly, EET-B treatment markedly improved the acetylcholine-mediated vasorelaxation in ANG II hypertensive rats and the mesenteric artery response to 10 μM acetylcholine averaged 78 ± 5% (*P* < 0.05). The mesenteric arteries response to a nitric oxide donor sodium nitroprusside were similar among the groups and averaged 93 ± 9%. These findings support the notion that mesenteric resistance artery vascular function in ANG II hypertension is partially preserved by EET-B treatment.

### EET-B PROTECTS THE KIDNEY IN ANG II HYPERTENSION

**FIGURE 4:**
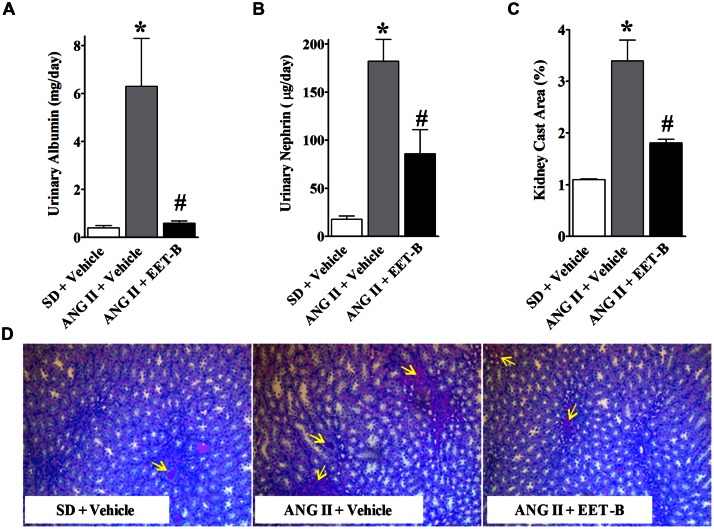
**Orally administered EET-B provides kidney protection in angiotensin II (ANG II) hypertension.** Urinary albumin **(A)**, and nephrin **(B)** excretion rates and formation of proteinacious cast in the kidney **(C,D)** were determined in SD, ANG II hypertension, and ANG II hypertensive rats treated with EET-B administered at a dose of 10 mg/kg/day in the drinking water for 14 days. *P < 0.05 vs. normal SD rat; #*P* < 0.05 vs. vehicle-treated ANG II hypertensive rat. All data presented as mean ± SEM, *n* = 6/group.

As demonstrated in **Figure 4**, EET-B treatment protected the kidney from ANG II hypertension-mediated damage. EET-B-treated ANG II hypertensive rats demonstrated lower urinary excretions of albumin and nephrin along with the presence of relatively normal renal histological features compared to vehicle-treated ANG II hypertensive rats. Indeed, marked albuminuria and nephrinuria were found in ANG II hypertensive rats and EET-B treatment protected the kidney as determined by 90% lower albuminuria and 54% lower nephrinuria (*P* < 0.05; **Figures 4A,B**). Moreover, in line with the marked albuminuria and nephrinuria in ANG II hypertensive rats, histopathological study revealed mild tubular proteinaceous cast formation, and these renal tubular casts were fewer in EET-B-treated rats (*P* < 0.05; **Figures 4C,D**).

### EET-B-TREATED ANG II HYPERTENSIVE RATS HAVE LOWER RENAL INFLAMMATION

**FIGURE 5:**
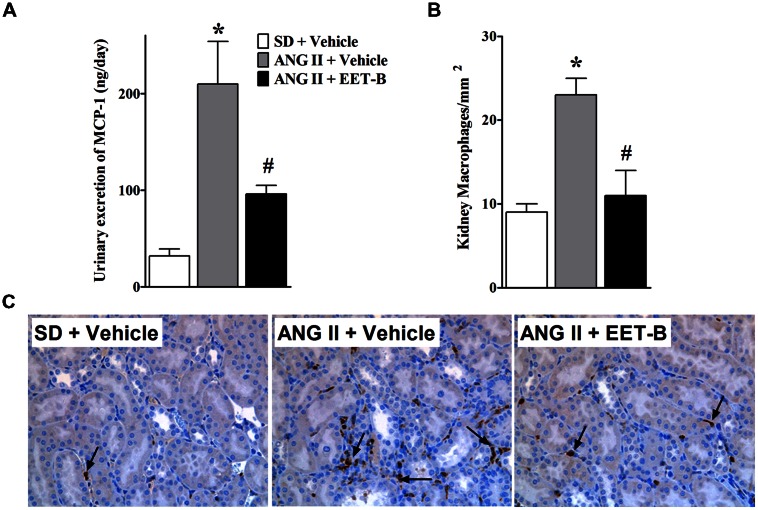
**EET-B reduced renal inflammation in angiotensin II (ANG II) hypertension.** Urinary MCP-1excretion **(A)** and kidney macrophage infiltration **(B,C)** were determined in SD, ANG II hypertension, and ANG II hypertensive rats treated with EET-B administered at a dose of 10 mg/kg/day in the drinking water for 14 days. *P < 0.05 vs. normal SD rat; #*P* < 0.05 vs. vehicle-treated ANG II hypertensive rat. All data presented as mean ± SEM, *n* = 6/group.

Angiotensin II hypertensive rats demonstrated renal inflammation with 80% higher urinary excretion of MCP-1 compared to control SD rats. Interestingly, EET-B-treated hypertensive rats had a55% lower renal MCP-1 excretion (**Figure 5A**). In line with markedly elevated MCP-1 excretion, we also observed a 60% increase in kidney infiltration of macrophages in ANG II hypertensive rats. EET-B-treated ANG II hypertensive rats had a 52% reduction in renal macrophage infiltration compared to vehicle-treated ANG II hypertensive rats (**Figures 5B,C**).

## DISCUSSION

Epoxyeicosatrienoic acids have a number of cardiovascular actions and play important roles in vascular and blood pressure control (Imig, 2010, 2012). Biological actions attributed to EETs are expected to provide organ protection in a number of pathologies including hypertension (Imig, 2010). In view of such important biological actions of EETs, there has been a growing interest in developing EET-based therapeutic strategies including the development of inhibitors of sEH to prevent degradation of EETs. Indeed, a large number of studies demonstrated beneficial effects of sEH inhibitors in a number of pathologies including hypertension (Imig et al., 2002, 2005; Zhao et al., 2004; Loch et al., 2007; Manhiani et al., 2009; Imig, 2010, 2012). On the other hand, sEH inhibitors have two drawbacks. First, they result in a generalized increase in EETs as well as other epoxides, and second, their effectiveness depends on epoxygenase-mediated EET generation (Imig and Hammock, 2009). This second limitation is important because renal and cardiovascular diseases are associated with impaired epoxygenase generation of EETs (Imig et al., 2002; King et al., 2005; Imig and Hammock, 2009; Imig, 2010). It is, therefore, likely that if epoxygenase-mediated EET generation is impaired, sEH inhibition will have a smaller or negligible effect on EET levels in these pathological conditions. As such, efforts have been made to develop stable EET agonist analogs with several key features important for their stability and bioavailability (Morisseau and Hammock, 2005; Falck et al., 2009). Several of these EET analogs have demonstrated cardiovascular actions in different experimental disease models (Imig et al., 2001, 2010; Seubert et al., 2007; Sodhi et al., 2009; Hye Khan et al., 2013, 2014; Khan et al., 2013). In the present study, we investigated anti-hypertensive, vascular, and kidney protective effects of one such EET analog, EET-B, in ANG II hypertensive rats. EET-B was designed by replacing acidic carboxyl group in carbon 1 of EET pharmacophore with a heterocyclic surrogate. We demonstrate that this EET analog attenuated the blood pressure elevation in ANG II hypertension when administered orally in the drinking water. Similar antihypertensive effects for EET analogs were reported in earlier studies with metabolic syndrome mice, spontaneously hypertensive rats and also recently in rats with ANG II hypertension (Sodhi et al., 2009; Imig et al., 2010; Hye Khan et al., 2014).

The pathophysiology of ANG II hypertension is associated changes in renal tubular sodium reabsorption and impaired vascular function (Fyhrquist et al., 1995; Jennings et al., 2012; Hye Khan et al., 2014). Vascular dysfunction constitutes an early independent predictor of cardiovascular events in hypertension and accordingly current therapeutic strategies are aimed at mitigating vascular dysfunction to reduce hypertension-related mortality and morbidity (Puddu et al., 2000; Perticone et al., 2001). Indeed, a larger reduction in the occurrence of cardiovascular events has been demonstrated in hypertensive patients when vascular function is restored (Perticone et al., 2001). Reduced EET levels are associated with vascular dysfunction in hypertension (Gao et al., 2011). In an earlier study, we demonstrated enhanced afferent arteriolar reactivity to ANG II in ANG II hypertension that was reversed by administering a sEH inhibitor to increase EET bioavailability (Imig et al., 2001). Moreover, a newly developed orally active EET analog, EET-A improved mesenteric resistance artery relaxation to acetylcholine in ANG II hypertension (Hye Khan et al., 2014). In accordance with these previous findings, the current study demonstrates that treatment with EET-B partially preserved acetylcholine-mediated dilation of mesenteric resistance arteries in ANG II hypertension. This vascular effect of EET-B could be a direct effect of EET-B on the vasculature. Indeed, in an earlier study, we demonstrated that EET-B caused marked vasodilation in pre-constricted mesenteric resistance artery and at that EET-B vasodilation was abolished in the presence of an EET antagonist, 14,15-epoxyeicosa-5(*Z*)-enoic acid (14,15-EEZE; Hye Khan et al., 2013). Thus past and current findings consistently demonstrate that sEH inhibitors and EET analogs including EET-B improve vascular function in hypertension.

In addition to ANG II vasoconstrictor actions, ANG II increases renal tubular sodium reabsorption which has been implicated in the pathophysiology of ANG II hypertension. ANG II hypertension is associated with sodium retention caused by increasing epithelial sodium channel (ENaC) activity and ENaC inhibition attenuates ANG II hypertension (Beutler et al., 2003; Gonzalez et al., 2011). Interestingly, EETs inhibit sodium reabsorption, and this biological activity is implicated in their ability to lower blood pressure (Imig, 2010, 2012). Indeed, EETs lower blood pressure by reducing tubular Na^+^ reabsorption via ENaC inhibition (Wei et al., 2004). ENaC inhibitory effects of EETs and EET analogs including EET-A have been demonstrated using electrophysiological measurements of the channel activity in immortalized mouse cortical collecting duct principal (mpkCCD_c14_) cells (Pavlov et al., 2011; Hye Khan et al., 2014). In contrast to these findings, using a similar electrophysiological approach, we recently reported that EET-B is a very weak ENaC inhibitor and did not affect basal Na^+^ transport in mpkCCD_c14_ cells (Hye Khan et al., 2014). In accord with the electrophysiological findings, EET-B did not affect sodium excretion in Dahl salt-sensitive hypertension (Hye Khan et al., 2013). In the current study, EET-B did not alter urinary sodium excretion as assessed on day 14 in ANG II hypertension. These data suggest that unlike EET-A, the anti-hypertensive action of EET-B does not involve significant changes in sodium reabsorption and is more likely more dependent on its vascular actions.

A pathophysiological consequence of hypertension-related mortality and morbidity is end organ damage. Accordingly, ANG II-dependent hypertension leads to kidney injury with marked proteinuria, albuminuria and renal histopathological changes (Jennings et al., 2012; Luft et al., 2012). Moreover, ANG II activates immune cells and promotes infiltration of such cells into organs including the kidney resulting in progressive renal injury (Park et al., 2009; Jennings et al., 2012). In this regard, EET and EET analog could play important roles in protecting from hypertension-mediated end organ damage. Indeed, a number of studies have demonstrated strong anti-inflammatory action of EETs and EET analogs that contributes to end organ protection in a number of preclinical models of human disease including hypertension (Olearczyk et al., 2009; Elmarakby et al., 2011; Imig, 2012; Hye Khan et al., 2013; Khan et al., 2013). In line with these previous findings, the present study also demonstrates marked kidney protective and anti-inflammatory effects of EET-B in ANG II hypertension along with a reduction in blood pressure. It is possible that in ANG II hypertension, the reductions in kidney injury and inflammation could be related to blood pressure reduction. However, in several earlier studies we have demonstrated that EET-B has blood pressure independent kidney protective and anti-inflammatory actions. In Dahl salt sensitive rats we have demonstrated that EET-B provides kidney protective as well as anti-inflammatory effects without affecting the blood pressure (Hye Khan et al., 2013). In another study with cisplatin-induced nephrotoxicity, we also have demonstrated that orally administered EET-B decreased renal injury and reduced renal mRNA expression of interleukin-6, interleukin-1β, and tumor necrosis factor-α (TNF-α) along with a marked reduction of renal TNF-α levels (Khan et al., 2013). Evidence for a direct anti-inflammatory action of EET-B was demonstrated in human umbilical vein endothelial cells where EET-B markedly attenuated the TNF-α induced inflammatory response (Hye Khan et al., 2013).Taken together, findings in earlier and the current study indicate that EET-B reduces renal inflammation and provides kidney protection in a number of pathologies including ANG II hypertension.

In summary, in ANG II hypertension, the EET analog EET-B reduces blood pressure elevation, partially preserves vascular function, and provides kidney protection with marked anti-inflammatory activity. These findings further demonstrate that the cardiovascular and renal actions of EET analogs make them a promising therapeutic approach for cardiovascular and renal diseases. Future development of EET-B and other EET analogs is required to determine their benefit to combat cardiovascular and renal diseases in humans.

## Conflict of Interest Statement

Drs. Imig, Falck, and Campbell have a patent application that covers the composition of matter for EET-B. There are no other conflicts of interest, financial or otherwise, are declared by the authors.
