# Erratum on: Epoxyeicosatrienoic acid analog attenuates angiotensin II hypertension and kidney injury

**DOI:** 10.3389/fphar.2014.00250

**Published:** 2014-11-19

**Authors:** Frontiers Production Office

**Affiliations:** Frontiers Production OfficeLausanne, Switzerland

**Keywords:** epoxyeicosatrienoic acid analog, mesenteric artery, endothelial function, renal inflammation, angiotensin II

Reason for erratum:

The name for the author Md. Abdul Hye Khan was listed as Abdul Hye Khan and the citation as Khan, AH instead of Hye Khan, MA, due to a typesetting error. This mistake does not change the scientific conclusions of the article in any way. The publisher apologizes for this error.

The original article has been updated.

